# Cyanosis of New-born Infants

**Published:** 1846-02-01

**Authors:** 


*Cyanosis of new-born Infants.*—Prof. Meigs, of Jefferson Medical College, Philadelphia, being in Paris at the sitting of the Academy of Sciences in June last, presented to that body a communication on the above subject. The following is the substance of the paper as reported in the Dublin Medical Press, and quoted in the Bulletin of Medical Science for September:

The remedy which Professor M. advises, the efficacy of which seems to be substantiated by an extensive experience in his hands, is exceedingly simple, and plausible. It should be borne in mind by every practitioner. He is of opinion that death from this affection results "from the presence of dark, non-oxygenated blood in the brain, where its presence is hurtful, not because it acts as a poison, but simply because it is incapable of exciting the motions of innervation in that organ. It is useless to insist on the universally known fact that the persistence of the foramen ovale is the cause of cyanosis. As the occlusion of the foramen ovale is prevented by the torrent of blood flowing from the inferior vena cava, raising and keeping raised the inter-auricular valve, which is thin and floating, it occurred to Dr. Meigs to place the cyanosed child on the right side, with the head and trunk somewhat raised, so that the inter-auricular septum should be maintained horizontal, and the blood contained in the left auricle should press with its whole weight on the closed valve. Dr. Meigs has frequently seen the blue color disappear at the very instant the infant was placed in this position, proving that oxygenated blood only entered the arteries.

Dr. Meigs stated that he had thus saved lives of fifty or sixty children in one hundred; whereas, as is well known, all the means hitherto tried have failed."

